# An integrated machine learning framework for developing and validating diagnostic models and drug predictions based on ulcerative colitis genes

**DOI:** 10.3389/fmed.2025.1571529

**Published:** 2025-06-13

**Authors:** Na An, Zhongwen Lu, Yang Li, Bing Yang, Shaozhen Ji, Xu Dong, Zhaoliang Ding

**Affiliations:** ^1^Shandong University of Traditional Chinese Medicine, Jinan, China; ^2^The Third Affiliated Hospital, Beijing University of Chinese Medicine, Beijing, China; ^3^Zibo City Fourth People’s Hospital, Zibo, China; ^4^Rizhao Hospital of Traditional Chinese Medicine, Rizhao, China

**Keywords:** ulcerative colitis, machine learning, immunity, molecular docking, dynamics, single cell, quantitative polymerase chain reaction

## Abstract

Ulcerative colitis (UC) is a long-lasting inflammatory bowel disease that causes inflammation in the intestines and triggers autoimmune responses. This study aims to identify immune-related biomarkers for ulcerative colitis (UC) and explore potential therapeutic targets. First, we downloaded the expression profiles of datasets GSE87466, GSE87473, and GSE92415 from the GEO database. Next, we identified differentially expressed genes (DEGs) that are associated with UC. Using the WGCNA algorithm, we screened key module genes in UC and retrieved immune-related genes (IRGs) from the ImmPort database. We identified immune-related differentially expressed genes by intersecting the results from WGCNA, DEGs, and IRGs. To build a diagnostic model for UC, we applied 113 combinations of 12 machine learning algorithms. This included 10-fold cross-validation on the training set and external validation on the test set. The single-cell results presented the cellular profile of UC and indicated that the key genes were significantly associated with macrophages, epithelial cells, and fibroblasts. The single-cell results presented the cell atlas of UC and suggested that key genes were significantly associated with macrophages, epithelial cells and fibroblasts. Quantitative polymerase chain reaction (q-PCR) was used to verify the expression levels of the core biomarkers screened out by machine learning. We conducted enrichment analysis using Gene Ontology (GO), Kyoto Encyclopedia of Genes and Genomes (KEGG), and gene set enrichment analysis (GSEA), which showed biological processes and signaling pathways associated with UC. Immune cell infiltration analysis based on CIBERSORT was also performed. We also screened potential drugs from the DSigDB drug database. To evaluate their effectiveness, we performed molecular docking and dynamics simulations. The results suggested that compounds like thalidomide and troglitazone are promising candidates for new UC drug development. Our findings provide insights into the pathogenesis of UC, its clinical treatment, and potential drug development.

## 1 Introduction

Ulcerative colitis (UC) is a specific chronic inflammatory disease of the colonic mucosa. The main symptoms of UC include frequent bowel movements, bloody diarrhea, abdominal pain, weight loss, and a strong urge to urinate (1, 2). These symptoms can increase the risk of developing colorectal cancer (3). UC is recognized as a worldwide health issue (4), and its prevalence has been increasing since the mid-20th century. It is projected that by 2035, more than 2 million individuals in Asia will be affected by UC (5, 6).

The effects of ulcerative colitis (UC) extend beyond intestinal lesions. Its complications may affect multiple systems throughout the body, posing a serious threat to patients’ health. According to literature reports, about 30% of UC patients will have extraintestinal manifestations, which are diverse and may even be life-threatening. For example, pulmonary embolism, sacroiliitis (7, 8), and other complications can arise, with toxic megacolon being particularly life-threatening (9). Furthermore, chronic inflammation over time significantly increases the risk of colorectal cancer. Among patients with extensive colitis, about 20% will develop cancer 30 years after the onset of the disease (10, 11). Additionally, about 15 to 30% of patients with acute severe UC eventually have to undergo colectomy (12).

Currently, existing treatment regimens have numerous limitations. Traditional preparations take effect slowly, and patients may be forced to discontinue medication due to adverse reactions. In the treatment of refractory UC, the exploration of combination therapy and new targeted drugs is still ongoing. Methotrexate, as an immunomodulator, only achieves complete remission in 42% of steroid-dependent patients, and 10% discontinue the medication due to side effects (13). In the combination therapy of basiliximab and hormones, although 50% of patients achieve remission within 8 weeks, 25% still require colectomy (14). In addition, drug metabolism-related gene polymorphisms significantly affect the efficacy of tacrolimus, with a remission rate of only 20% in patients carrying the TT genotype, highlighting the importance of individualized medication (15). These data collectively reveal that current treatment regimens still need further optimization in terms of sustained remission, safety, and individualized precision treatment.

The pathogenesis of UC is complex and not fully understood, involving multiple contributing factors. It involves the interaction of multiple factors. Among these factors, the immune mechanism is particularly significant in the development of UC. Research indicates that an imbalanced mucosal immune response significantly contributes to the development and worsening of UC (3, 16). Immune cells, particularly monocytes and lymphocytes, regulate inflammatory responses by secreting cytokines and chemokines, thereby affecting the gut microenvironment and immune status. For instance, the NLRP3 inflammasome is considered an important therapeutic target for UC, as its activation can lead to the exacerbation of inflammation (17). Therefore, targeted treatments for specific immune cells, such as the application of regulatory T cells (Treg), may help restore immune balance and reduce inflammation (18). In conclusion, the immune mechanisms underlying ulcerative colitis are complex and dynamic, involving numerous immune cells and cytokines. Further exploration of the relationship between these elements can provide new insights for the early diagnosis and treatment of UC.

**Figure 1:**
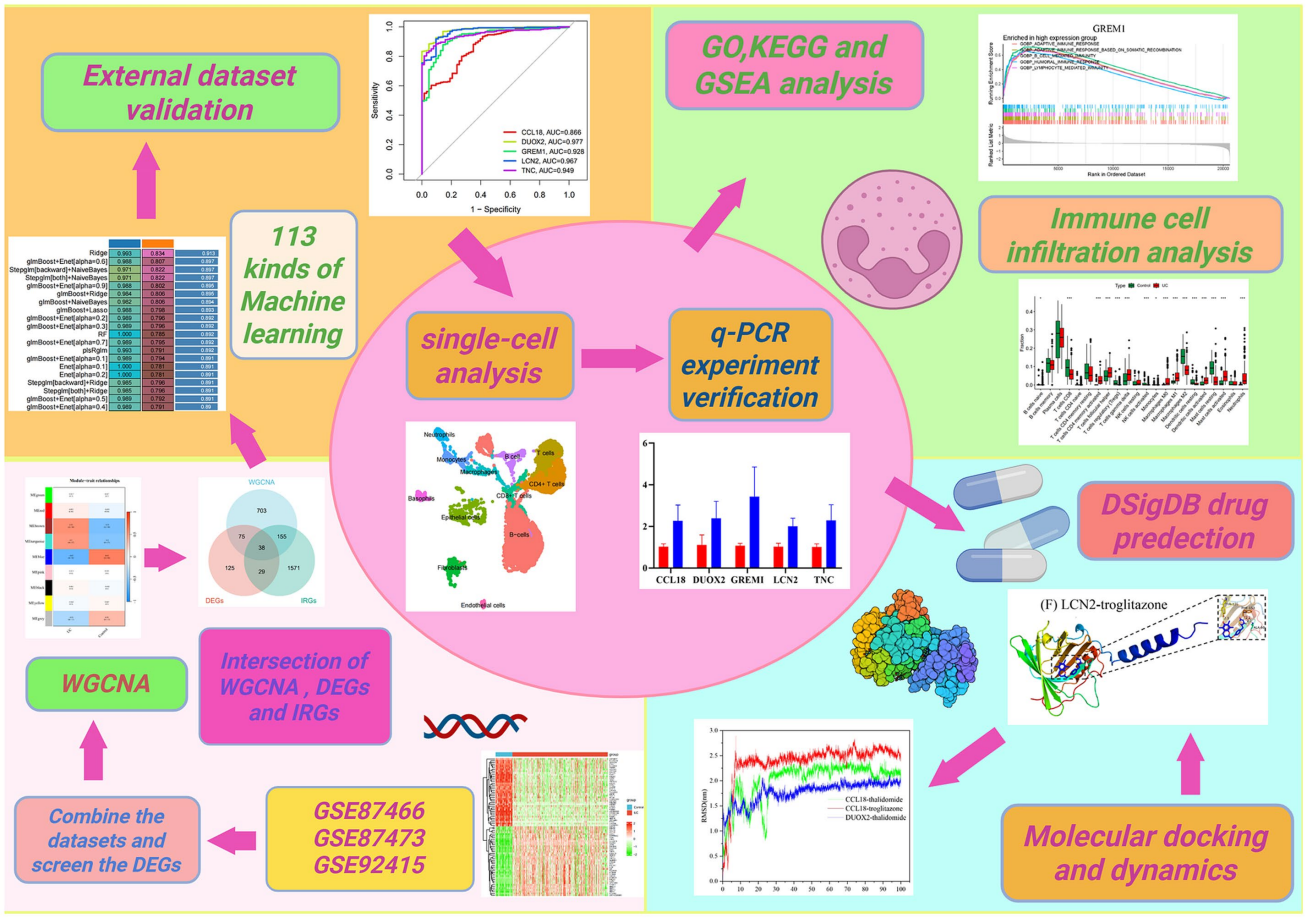
Flow chart of this study.

Recently, advancements in computer technology have made microarray technology and bioinformatics increasingly important in medicine. Powerful algorithms can create diagnostic models from high-throughput data to predict diseases. This method effectively combines large-scale genomic data with advanced analytical techniques to identify core biomarkers for disease diagnosis and treatment (19, 20). This study employed an integrated bioinformatics approach, analyzing expression profiles from the GEO database to investigate the molecular basis of UC, aiming to identify differentially expressed genes (DEGs). Weighted gene co-expression network analysis (WGCNA) was applied, which helps in identifying key gene modules associated with UC. The study aimed to identify immune-related DEGs by intersecting DEGs, WGCNA with immune-related genes, thus discovering potential biomarkers for UC. Furthermore, the study utilized a robust machine learning framework, exploring 12 algorithms among 113 combinations to construct a diagnostic model for UC. It also conducted Gene Ontology (GO) and Kyoto Encyclopedia of Genes and Genomes (KEGG) analyses to elucidate disease-related biological pathways. Additionally, the CIBERSORT algorithm was used to assess immune cell infiltration, and the DSigDB drug database was employed to identify potential therapeutic drugs. Subsequently, molecular docking and dynamic simulations were conducted, providing deeper insights into the pathogenesis and potential treatment strategies for UC. The research workflow is illustrated in Figure 1.

## 2 Results

### 2.1 Identification of DEGs in ulcerative colitis tissue

**Figure 2:**
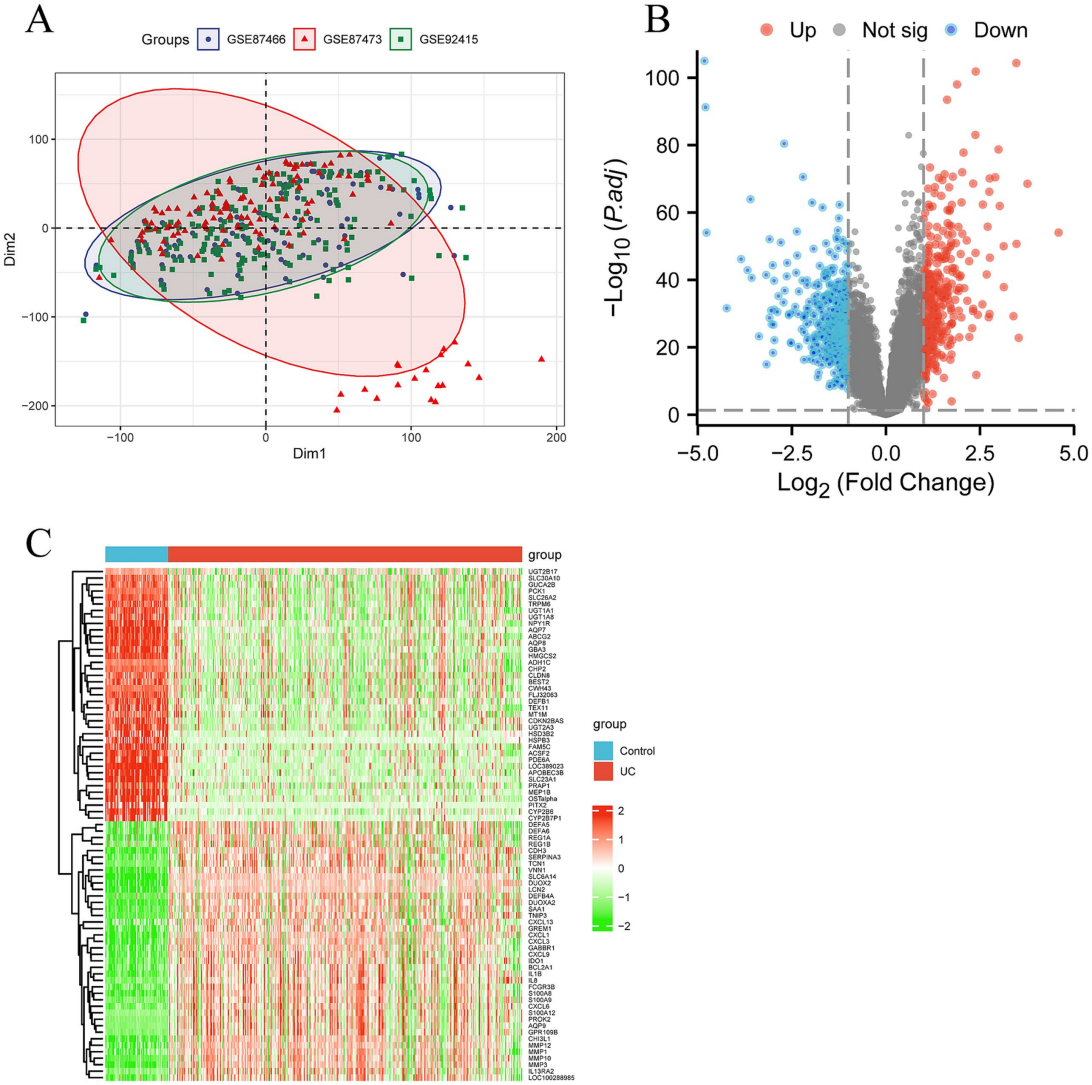
Screening differentially expressed genes (DEGs) and senescence related DEGs in ulcerative colitis (UC). **(A)** The principal component analysis (PCA) displaying a distinct profile between GSE87466, GSE87473 and GSE92415. **(B)** The volcano plot showing upregulated (red) and downregulated (green) DEGs. **(C)** Clustering analysis and heatmap of the DEGs between UC and control groups.

Data downloading and processing were obtained from the GEO database (Gene Expression Omnibus), which included three original datasets (GSE87466, GSE87473, and GSE92415). GSE87466 is based on the GPL13158 platform and includes 87 UC patient samples and 21 normal (control) samples; GSE87473 is also based on the GPL13158 platform and includes 106 UC patient samples and 21 control samples; GSE92415 is based on the GPL13158 platform and includes 162 UC patient samples and 21 control samples. After preprocessing and removing batch effects (Figure 2A), a total of 267 differentially expressed genes (DEGs) were obtained using |logFC| >1 as the criterion, including 114 upregulated genes and 153 downregulated genes in UC samples. The volcano plot is shown in Figure 2B, and the heatmap is shown in Figure 2C.

### 2.2 Construction and module identification of WGCNA co-expression network and acquisition of immune genes

**Figure 3:**
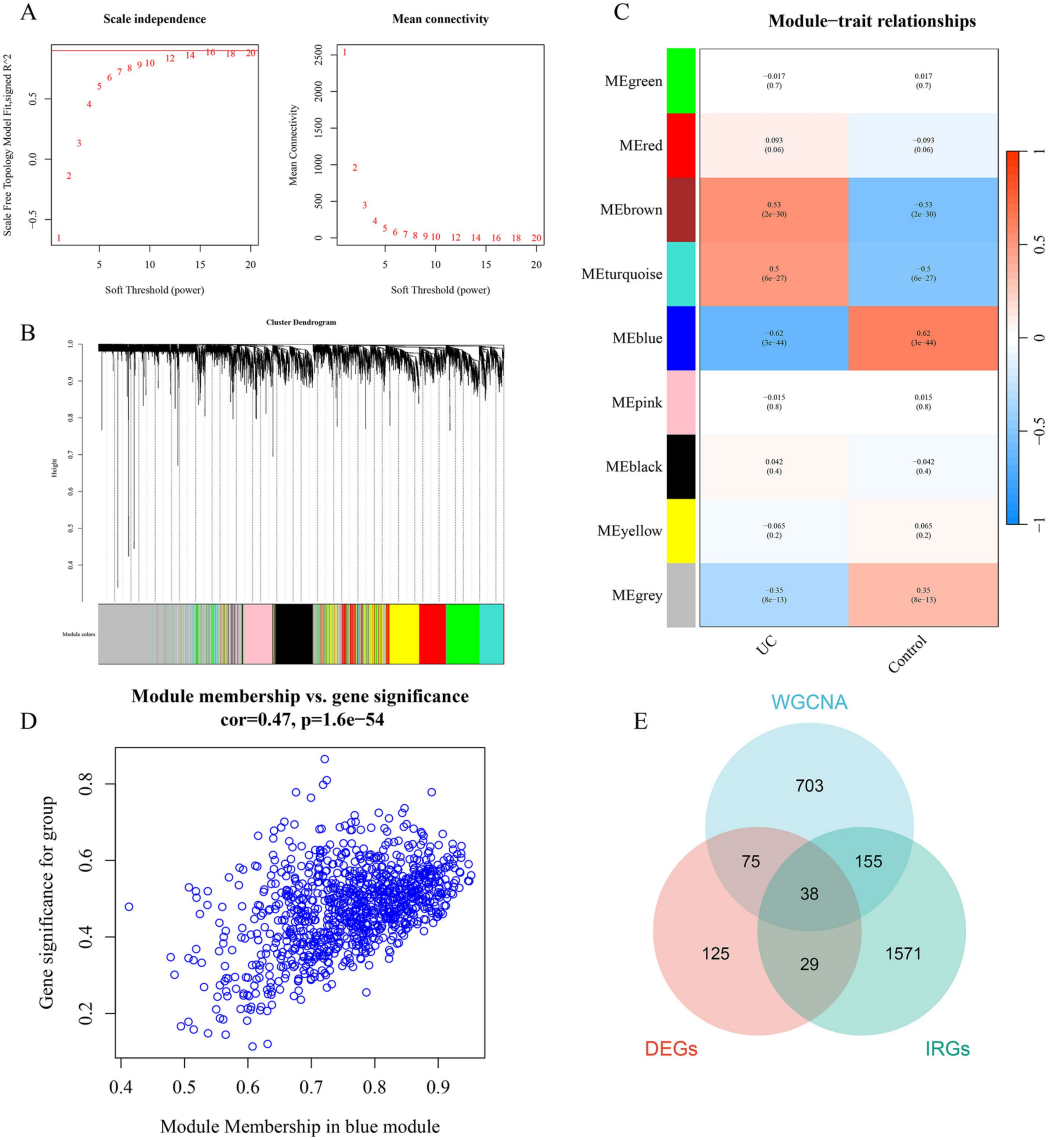
Identification of gene modules associated with UC using WGCNA. **(A)** The selection of optimal soft thresholding power. **(B)** Gene dendrogram and modules. Gene modules associated with UC were shown in different colors under the gene dendrogram. **(C)** The correlation heatmap representing the relationship between different gene modules and status of DFU. **(D)** Scatter plot showing the correlation between module membership (MM) and gene significance (GS) in the blue module. **(E)** Intersection between DEGs, IRGs, and WGCNA. WGCNA, weighted gene co-expression network analysis.

Using the R package “WGCNA,” a gene co-expression network analysis was constructed. First, clustering of samples was performed to ensure the accuracy of subsequent analyses. To ensure that the interactions between genes maximally conform to a scale-free distribution, the determination of soft-thresholding was carried out. As shown in Figure 3A, the optimal soft-threshold was determined to be 14 based on calculations from R software, at which point the *R*^2^ value on the vertical axis of Figure 3A (left) is close to 0.9. Based on the optimal soft-threshold, gene modules in different DFUs were identified using the hybrid dynamic branch cutting algorithm (Figure 3B). The top 10,000 genes ranked by variation were clustered and merged into 9 co-expression modules (Figure 3C). Pearson correlation analysis was used to explore the correlation between module characteristic genes and clinical traits. The results showed that the correlation of each module with clinical features was generally moderate, with the blue module (cor = 0.47, *p* < 0.01) showing a higher correlation than other modules, as seen in Figure 3C. Therefore, this module was selected as the important module, with a total of 971 genes for subsequent analysis (Figure 3D). ImmPort is one of the most authoritative human immune gene databases, allowing for the download of data for effective analysis. From the ImmPort database,^1^ a set of human immune genes was downloaded and after removing duplicate genes, 1,793 IRGs were obtained. Taking the intersection of the DEGs, WGCNA blue module genes, and IRGs, a total of 38 genes were obtained (Figure 3E).

### 2.3 GO function and KEGG pathway enrichment analysis

**Figure 4:**
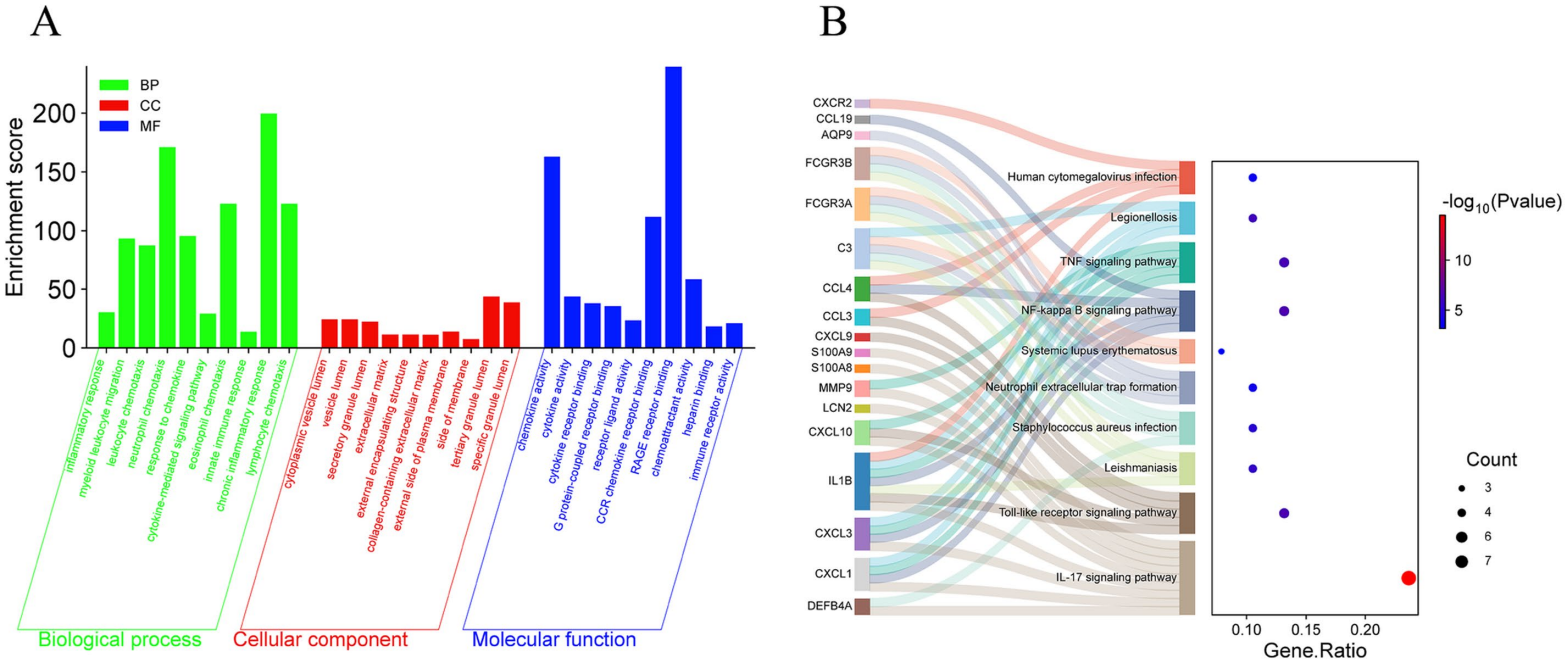
GO and KEGG enrichment analysis of DFU. **(A)** The bar chart of the GO enrichment of DEGs, including biological process, cellular component, and molecular function. **(B)** The Sankey diagram showing the KEGG enrichment analysis of DEGs.

Upload 38 differential genes to the Metascape database for GO and KEGG enrichment analysis, set the filtering condition to *p* < 0.01, and a total of 314 GO pathways and 27 KEGG pathways were obtained. Using −log_10_ (*p*-value) as the filtering condition, the top 10 annotation results of biological processes, cellular components, and molecular functions in the GO pathways and the top 10 KEGG pathways were selected to create a bubble chart. The biological processes mainly involve inflammatory response, myeloid leukocyte migration, leukocyte chemotaxis, etc. The cellular composition mainly involves the extracellular matrix, external encapsulating structure, lysosome, etc.; molecular function mainly involves chemokine activity, cytokine activity, cytokine receptor binding, etc. (Figure 4A); KEGG pathways (Figure 4B) mainly involve the IL-17 signaling pathway, Toll-like receptor signaling pathway, NF-kappa B signaling pathway, etc.

### 2.4 Identify hub genes with diagnostic value through machine learning and develop a diagnostic model for UC

**Figure 5:**
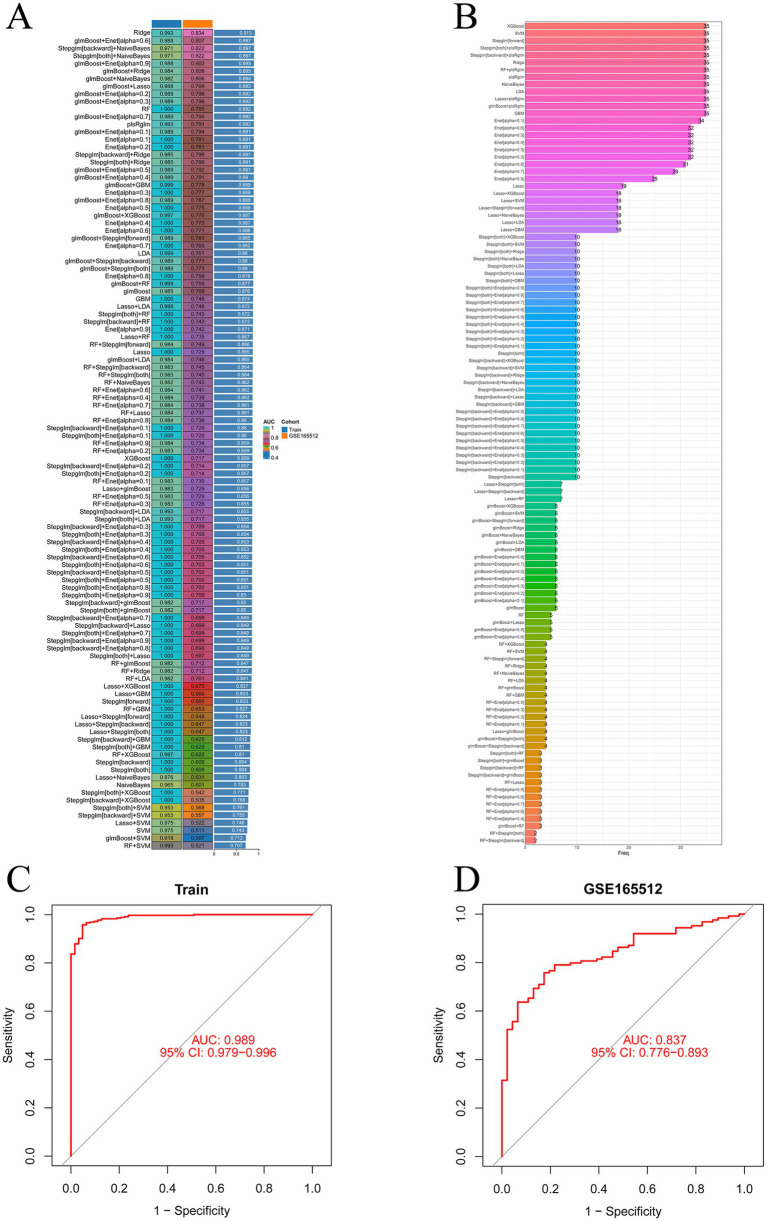
Machine learning prediction models. **(A)** One hundred and thirteen machine learning algorithm combinations evaluated via 10-fold cross-validation. **(B)** Number of genes selected by machine learning algorithm models. **(C)** AUC value of the training set. **(D)** AUC value of the validation set.

During the 10-fold cross-validation process, a total of 12 machine learning algorithms were combined to determine the most powerful diagnostic model based on five shared genes. The AUC values of the models and the number of genes selected are shown in Figures 5A,B. The algorithm Rige with the highest AUC value identified 35 genes, while the second-best algorithm glmBoost + Enet [alpha = 0.6] identified 5 genes (CCL18, DUOX2, GREM1, LCN2, and TNC). These five genes were included among the 35 identified genes. The AUC values of the two algorithms differed by only 0.016. To make the model calculation faster, achieve higher positive benefits, and receive less interference, this approach was adopted, the five genes selected by the glmBoost + Enet [alpha = 0.6] algorithm were chosen for further analysis. The AUC value of the training set was 0.989 (Figure 5C), and the AUC value of the validation set GSE165512 was 0.791 (Figure 5D), both demonstrating good predictive rates. This indicates a high level of consistency between the predicted probabilities derived from the model and the actual observed clinical outcomes, thereby demonstrating robust calibration performance.

### 2.5 Evaluating machine learning models

**Figure 6:**
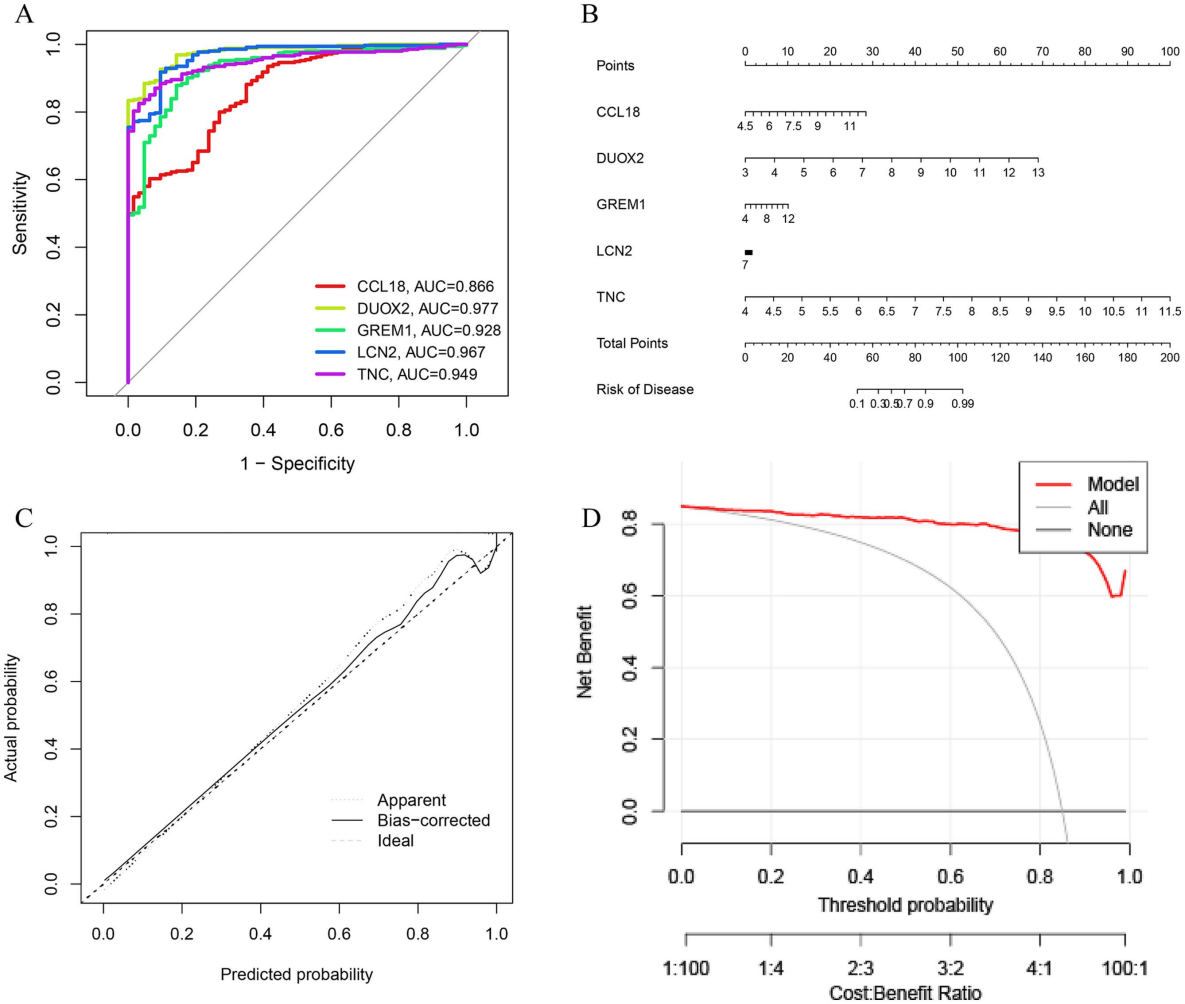
Validation based on differentially expressed associated genes (DEGs). **(A)** ROC curve of seven feature genes. **(B)** The diagnostic nomogram based on seven feature genes. **(C)** Calibration curve to evaluate the accuracy of the nomogram. **(D)** Decision curve of feature genes nomogram.

Use the glmBoost + Enet [alpha = 0.6] algorithm to select five genes (CCL18, DUOX2, GREM1, LCN2, and TNC) for validation, we obtained scoring scales for these seven feature genes individually, the performance of genes in each model was quantitatively evaluated by their AUC values, which were displayed in the ROC curve (Figure 6A). We summed the feature gene expression scores to the line plot (Figure 6B), assessed the relevancy associated with the feature genes related to immunization in relation to UC. Furthermore, the calibration curve shows the minimum error between the actual DFU cluster risk and the predicted risk (Figure 6C). The DCA and calibration curve were used to evaluate the predictive accuracy of the line plot model. DCA demonstrates that the line plot has high accuracy and can provide reference for clinical decision-making (Figure 6D).

### 2.6 Single-cell analysis

**Figure 7:**
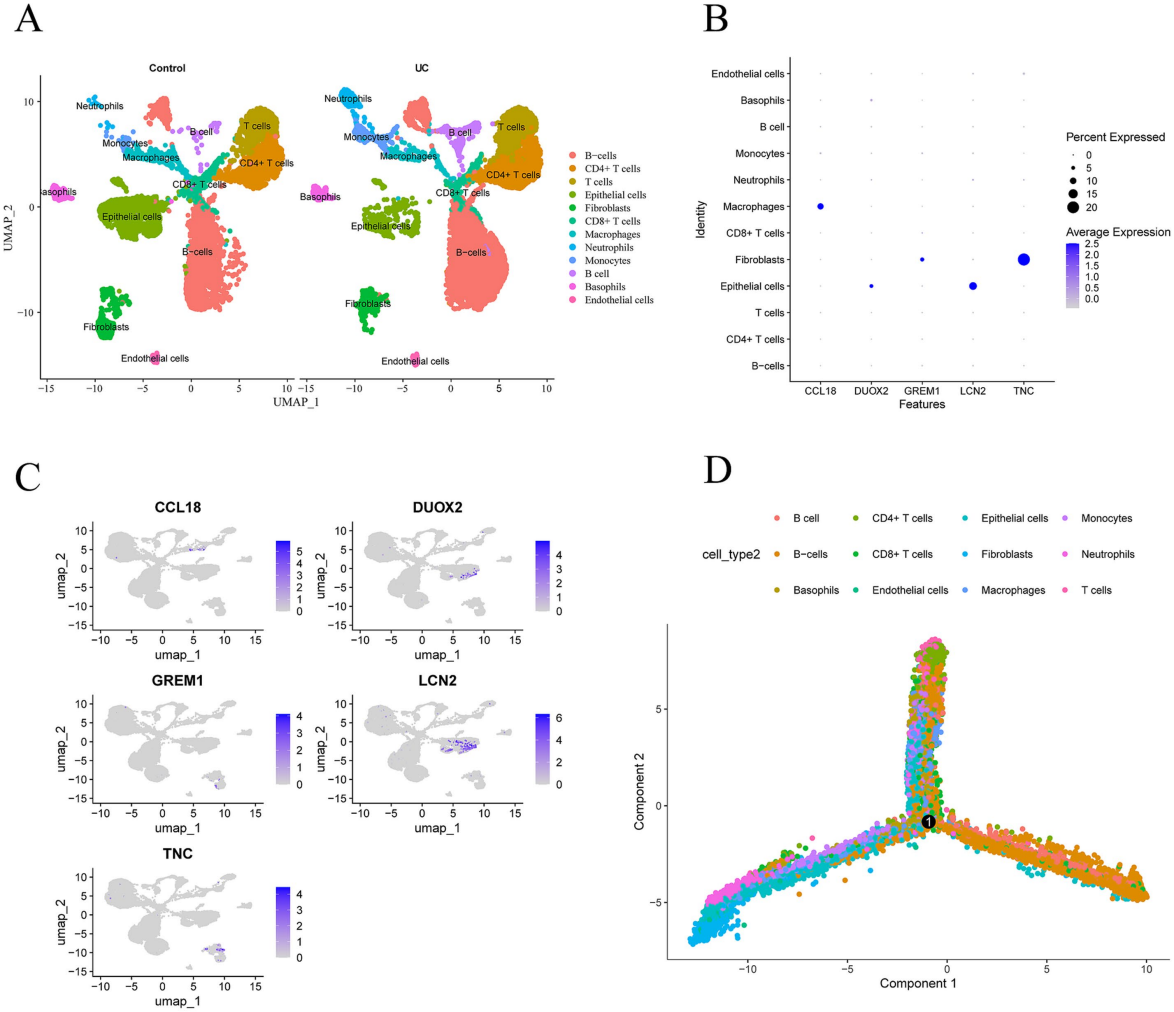
Expression profiles of hub genes in single cells. **(A)** Cellular subtypes of UC. **(B,C)** Scatter plots and bubble plot of the expression of the five hub genes. **(D)** Cell trajectory diagrams of twelve types of cells.

Download the single-cell data of GSEE214695 from the GEO database and conduct single-cell analysis through the Seurat package. Utilize the tSNE algorithm and UMAP (uniform manifold approximation and projection) to cluster the cells. Then, use the R package SingleR to annotate each cluster. All cells are categorized into 12 categories: B-cells, CD4^+^ T cells, CD8^+^ T cells, epithelial cells, fibroblasts, macrophages, neutrophils, monocytes, basophils, and endothelial cells (Figure 7A). The expression levels of CCL18, DUOX2, GREM1, LCN2, and TNC in these 12 types of cells are shown in Figures 7B,C. The five core genes are most significantly expressed in three cell types: macrophages, fibroblasts, and epithelial cells. Among them, CCL18 is significantly expressed in macrophages, DUOX2 and LCN2 are most significantly expressed in epithelial cells, and GREM1 and TNC are most significantly expressed in fibroblasts. Figure 7D shows the distribution and potential differentiation trajectories of these 12 types of cells in ulcerative colitis samples through dimensionality reduction analysis, revealing the dynamic changes and interrelationships among cells under the UC state.

### 2.7 q-PCR validation of core genes expression

**Figure 8:**
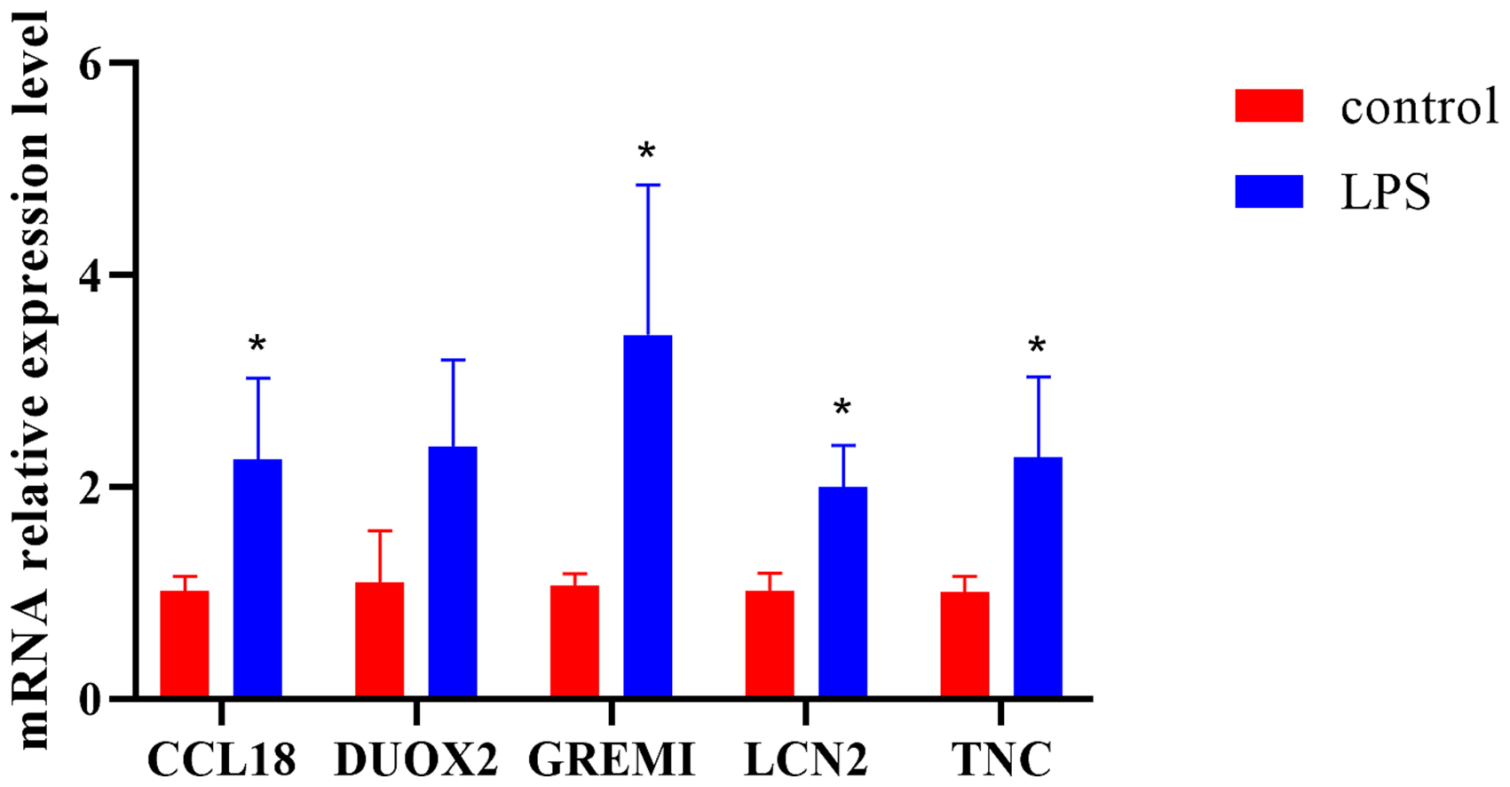
The q-PCR analysis results of key gene expression. ^*^p < 0.05.

The expression levels of core immune genes including CCL18, DUOX2, GREM1, LCN2, and TNC in ulcerative colitis models were detected by fluorescence q-PCR. The experimental results showed that compared with the normal control group, the expression levels of CCL18, DUOX2, GREM1, LCN2, and TNC in the LPS group were all upregulated, among which the upregulation of GREM1 was the most prominent and there was a significant difference from the normal group. Therefore, it can provide guidance for the diagnosis and treatment of UC (Figure 8).

### 2.8 Gene set enrichment analysis

**Figure 9:**
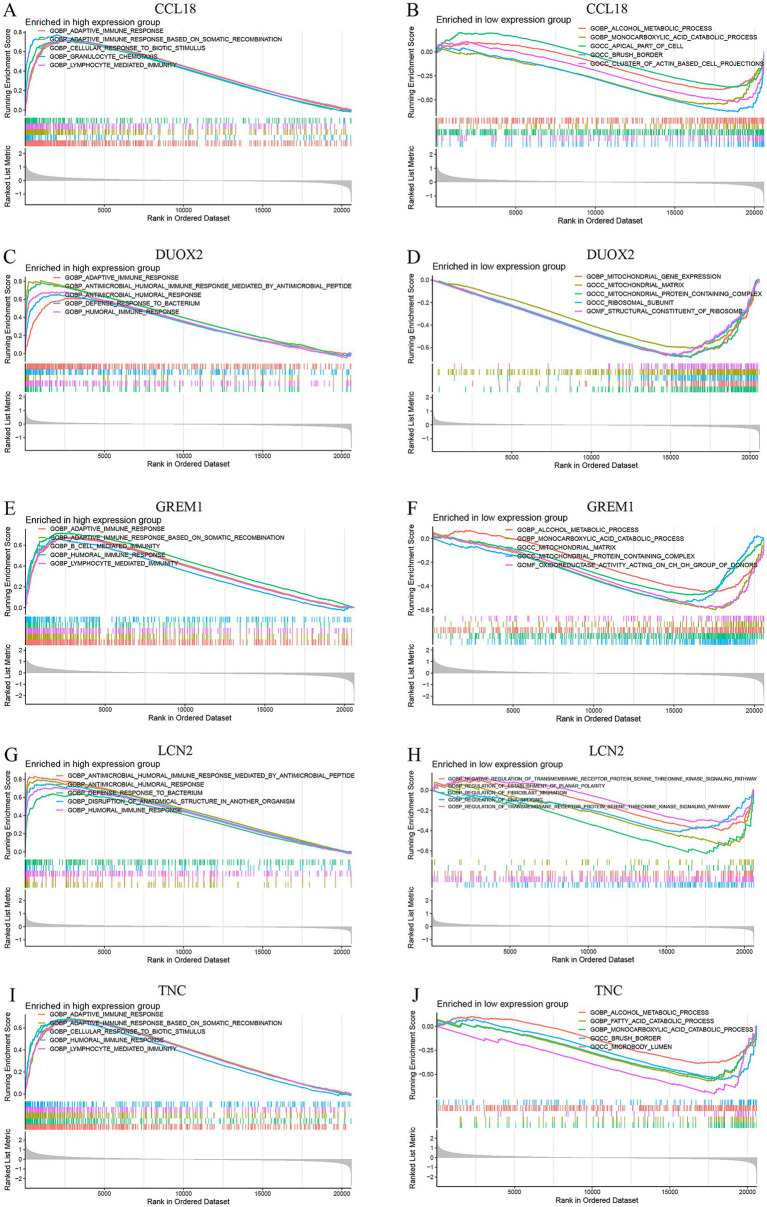
GSEA enrichment analysis conducted on the CCL18, DUOX2, GREM1, LCN2 and TNC genes, including enrichment in both high-expression and low-expression groups. (**A**, **C**, **E**, **G**, **I**) show the enrichment results of the high-expression groups of the CCL18, DUOX2, GREM1, LCN2 and TNC genes, while (**B**, **D**, **F**, **H**, **J**) present the enrichment results of the low-expression groups of these genes.

The results of the GSEA enrichment analysis show that these markers are involved in multiple pathways related to the occurrence and development of DFU, mainly involving myeloid leukocyte migration, granulocyte migration, epidermal cell differentiation, intermediate filament organization, keratinocyte differentiation, extracellular matrix structural constituent, collagen catabolic process, epidermis development, neutrophil chemotaxis, skin development, specific granule membrane, secretory granule membrane, antimicrobial humoral response, detection of stimulus involved in sensory perception, collagen containing extracellular matrix, receptor complex, translation factor activity RNA binding, tertiary granule. All these pathways may be closely related to immune regulation in DFU (Figure 9).

### 2.9 Immune infiltration

**Figure 10:**
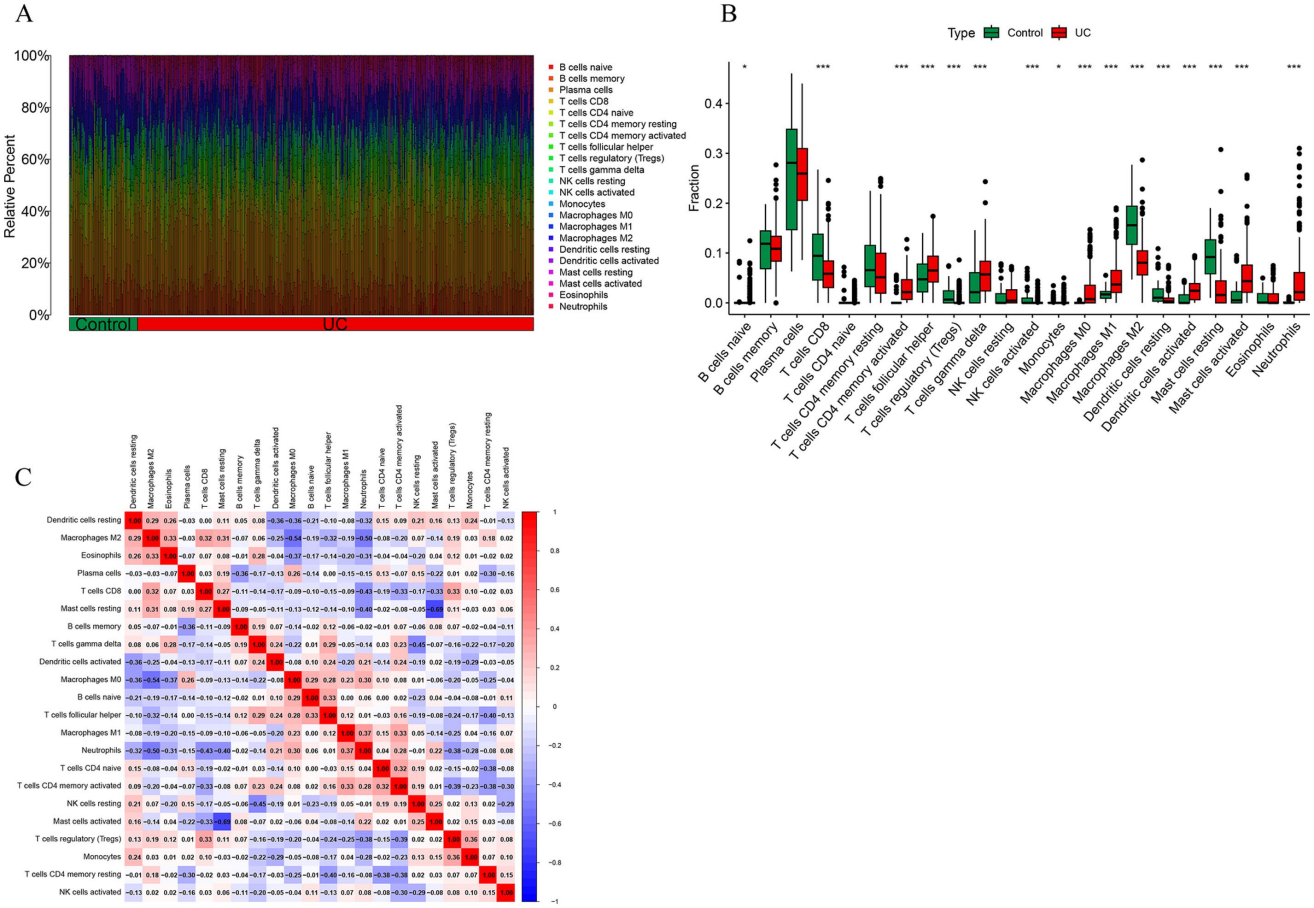
Immune cell infiltration analysis. **(A)** The stacked bar plot representing the different immune cell proportions in each sample. **(B)** The heatmap showing the correlation between different immune cells. Red represented a positive correlation, while blue represented a negative correlation. **(C)** The boxplot depicting the comparison of 22 types of immune cells between DCM and control groups.

The immune abundance of UC patients and controls was evaluated using the CIBERSORT algorithm, the barplot was generated to visualize the proportions of immune cells in each sample, as shown in Figure 10A. The immunodifference analysis chart Figure 10B shows the comparison between UC patients and normal tissues, the proportion of macrophages M0, macrophages M0, mast cells activated and neutrophils were higher compared with control group. And the proportion of T cell CD8, macrophages M2 and mast cells resting were lower compared with control group, as shown in Figure 10B. In addition, the correlation analysis of 22 types of immune cells indicated that the correlation between monocytes and T cells regulatory (Tregs) (*r* = 0.36, *p* < 0.05) showed positive correlation. Correlation between mast cells activated and mast cells resting (*r* = 0.69, *p* < 0.05), macrophages M0 and macrophages M2 were negatively associated, as shown in Figure 10C.

### 2.10 Identification of candidate drugs

Model genes were analyzed using the DSigDB drug database on Enrichr to identify potential targeting agents. Top 10 drug candidates were beclomethasone, ibuprofen, glycoprotein, simvastatin, methotrexate, prednisolone, budesonide, troglitazone, sulfasalazine and thalidomide (Table 1).

**Table 1:** Predicted drug information.

Term	*p*-value	Combined score	Genes
Beclomethasone	2.85195 × 10^−9^	1203.557661	CXCL10; IL1B; CCL4; CCL3; MMP9; LTF
Ibuprofen	1.14966 × 10^−8^	576.1780299	C3; CXCL10; IL1B; TNC; CCL3; DEFB4A; MMP9
Glycoprotein	5.93118 × 10^−8^	408.3725055	IL1B; TNC; LCN2; STC1; PI3; MMP9; LTF
Simvastatin	8.55094 × 10^−8^	288.3396864	MMP12; IL1B; CCL4; CCL3; MMP9; S100A9; S100A8; IDO1
Methotrexate	1.62111 × 10^−7^	177.4477246	C3; FCGR3B; IL1B; CCL4; CXCR2; TNC; CCL3; PI3; CCL19; S100A9
Prednisolone	1.86306 × 10^−6^	719.0071486	IL1B; CCL4; CXCR2; CCL3
Budesonide	2.59611 × 10^−6^	641.1431892	IL1B; CCL4; CCL3; LTF
Troglitazone	2.71535 × 10^−6^	119.6986126	CXCL10; IL1B; IL24; CCL4; CCL3; STC1; CXCL1; PI3; MMP9
Sulfasalazine	4.67698 × 10^−6^	522.6005686	IL1B; CXCL1; MMP9; IDO1
Thalidomide	6.75068 × 10^−6^	262.9742949	C3; IL1B; CXCR2; MMP9; LTF

### 2.11 Molecular docking

**Figure 11:**
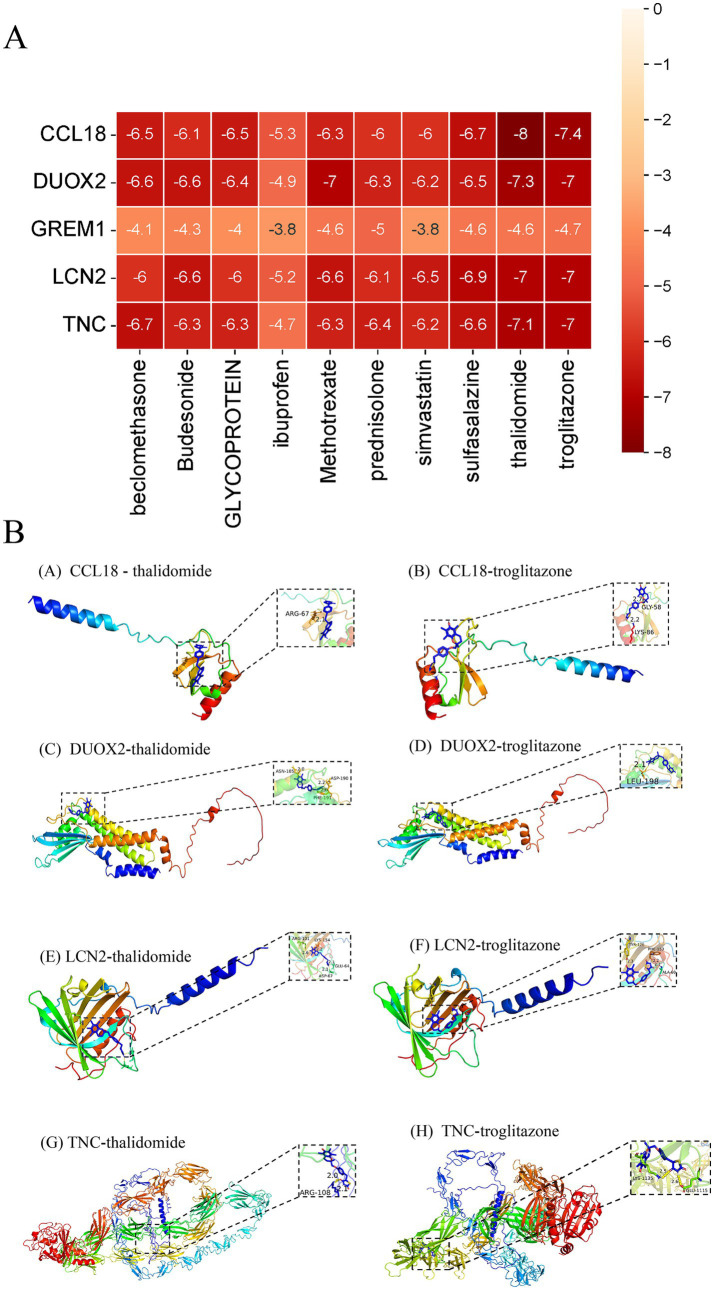
Results of molecular docking. **(A)** Binding energy results of molecular docking. **(B)** Presentation of molecular docking results.

According to the receptor-ligand docking theory, the docking energy is inversely proportional to the binding affinity; the more negative the docking energy, the stronger the binding affinity between the protein and the ligand. The results of molecular docking as shown in Figure 11A. The eight compounds and proteins with the best binding affinity are: CCL18-thalidomide, CCL18-troglitazone, DUOX2-thalidomide, DUOX2-troglitazone, LCN2-thalidomide, LCN2-troglitazone, TNC-thalidomide and TNC-troglitazone. Import the docking data of the above eight pairs of compounds and proteins into Pymol software for visualization to analyze the interactions between compounds and proteins, as shown in Figure 11B. Eight central therapeutic targets and the binding energy values of active compounds acting on them are all less than −5 kcal/mol, and they form at least one hydrogen bond with each other, indicating that these compounds have good binding affinity with key therapeutic targets and can fully exert their anti-DFU effects. The selection criteria for the core active ingredients are as follows: (1) compounds with the highest affinity for each key therapeutic target; (2) compounds acting on key therapeutic targets must have a docking energy of less than −5 kcal/mol. According to the above selection criteria, the key active ingredients for treating UC are thalidomide and troglitazone.

### 2.12 Molecular dynamics analysis

**Figure 12:**
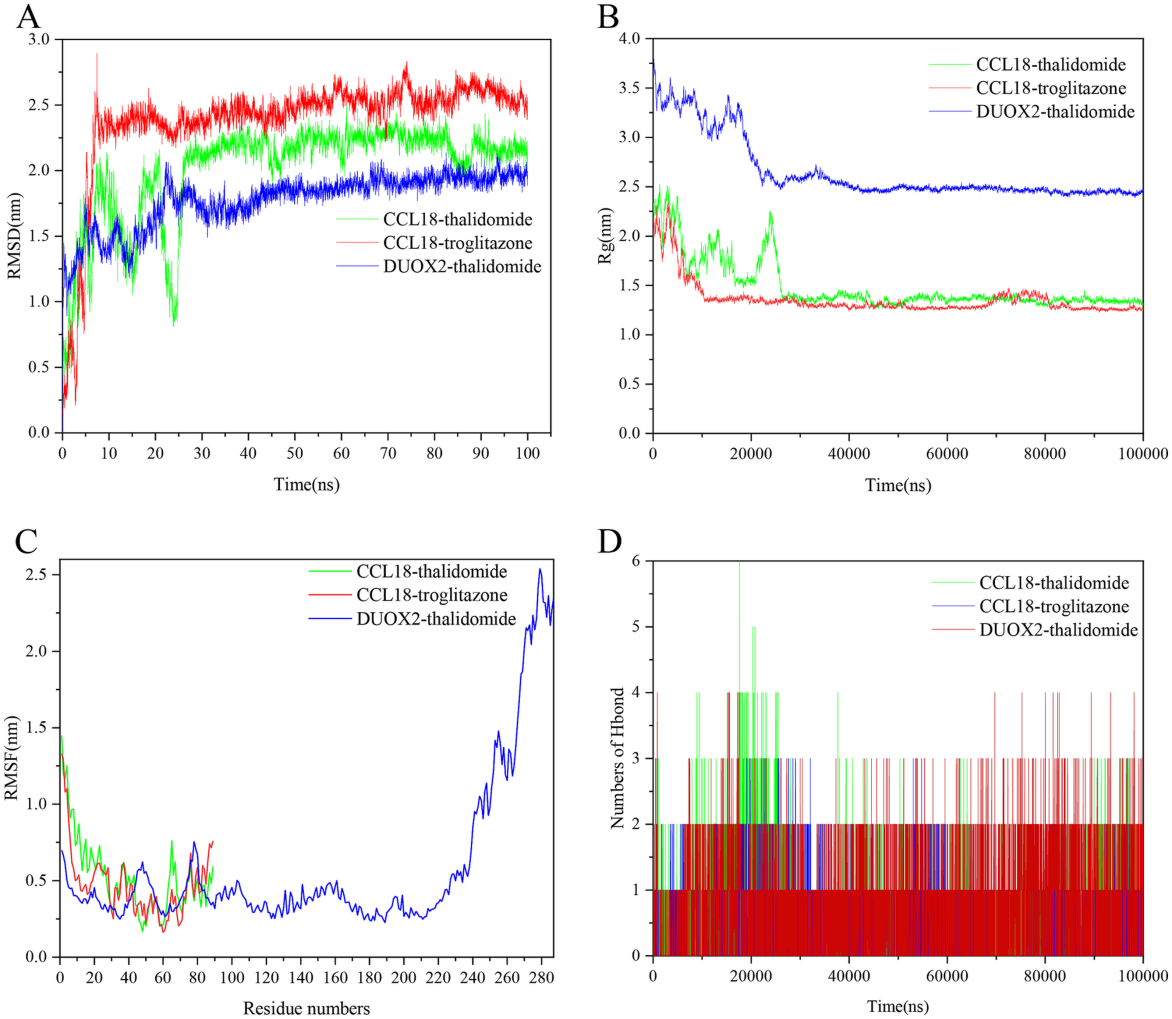
The molecular dynamics (MD) simulation of the PLA2G2A and naftopidil complex, PLA2G2A and PP-30 complex and FGFR3 and AZD-8055 complex. **(A)** The RMSD plot of the PLA2G2A and naftopidil complex, PLA2G2A and PP-30 complex and FGFR3 and AZD-8055 complex. **(B)** The Rg plot of the PLA2G2A and naftopidil complex, PLA2G2A and PP-30 complex and FGFR3 and AZD-8055 complex. **(C)** The RMSF plot of the PLA2G2A and naftopidil complex, PLA2G2A and PP-30 complex and FGFR3 and AZD-8055 complex. **(D)** The number of hydrogen bonds in the PLA2G2A and naftopidil complex, PLA2G2A and PP-30 complex and FGFR3 and AZD-8055 complex.

As shown in Figure 12A, the combinations of protein CCL18 with compounds thalidomide, protein CCL18 with compound troglitazone, and protein DUOX2 with compound thalidomide exhibit good binding activity and docking stability. These three combinations were selected for kinetic simulation. From the graph, it can be seen that the root mean square deviation (RMSD) of the small molecules remains stable, while the RMSD of the complexes and proteins shows initial fluctuations, becomes stable in the middle, and experiences minor fluctuations towards the end (Figure 12A). As can be seen from Figure 12B, the radius of gyration (Rg) of the complex is stable, indicating that the overall structure of the complex is compact and robust. The root mean square fluctuation (RMSF) can be used to represent the fluctuation of the complex at the residue level. From Figure 12C, it can be seen that proteins DUOX2 and compounds thalidomide have greater residue flexibility. Hydrogen bonding is a strong non-covalent interaction. The number of hydrogen bonds in the CCL18 and thalidomide was 0-6, CCL18 and troglitazone was 0–3, DUOX2 and thalidomide was 0–4, as shown in Figure 12D. The hydrogen bond between the ligand and the receptor helps maintain the stability of the complex.

## 3 Method

### 3.1 Data download and processing

We obtained four raw datasets (GSE87466, GSE87473, GSE92415, and GSE165512) from the GEO database (Gene Expression Omnibus). GSE87466 is based on the GPL13158 platform, including 87 UC patient and 21 normal (control) samples; GSE87473 is based on the GPL13158 platform, including 106 UC patients and 21 control samples; GSE92415 is based on the GPL13158 platform, including 162 UC patients and 21 control samples; GSE165512 is based on the GPL16791 platform, including 124 UC patients and 46 control samples. Subsequently, probes in each dataset were annotated and converted to standard gene names based on the corresponding platform annotation files. We combined the expression profiles from the GSE87466, GSE87473, and GSE92415 datasets into a training set. We then adjusted the batch effects using the sva package, preparing the data for subsequent WGCNA and machine learning analyses. Additionally, GSE165512 was used as an additional dataset for validation. After standardization and normalization of the datasets, the “limma” R package was used to analyze the differentially expressed genes between DFU and control samples. The screening criteria were (|logFC| >1, *p* < 0.05). Volcano maps and heat maps were drawn using the R software ggplot2 package and the “ComplexHeatmap” package, respectively, to display the significantly regulated important genes.

### 3.2 Acquisition of immune-related genes

ImmPort is one of the most authoritative human immune gene databases, from which data can be downloaded for effective analysis. Download the human immune gene set from the ImmPort database (see text footnote 1), after removing duplicate genes, a total of 1,793 immune-related genes (IRGs) are identified.

### 3.3 Weighted gene co-expression network analysis and identification of core modules

WGCNA is a commonly used modular analysis technique often used to identify and screen biomarkers or drug targets for complex diseases, to identify highly co-varying gene expression matrices, and to provide new directions for studying the potential mechanisms of diseases. A co-expression network was constructed using the “WGCNA” package in R software (21). A co-expression network was built using the top 10,000 genes with the highest variance from the merged dataset, with the minimum number of genes within a module set to 100. Subsequently, we transformed the adjacency matrix into a topological overlap matrix (TOM) and constructed multiple gene modules and hierarchical clustering trees. Finally, the correlation between each module and clinical characteristics was assessed, and gene significance (GS) and module membership (MM) were calculated to measure the correlation and significance of genes with biological modules and clinical information, extracting important modules and genes for subsequent analysis. The intersection of immune-related genes, differentially expressed genes, and WGCNA module genes yielded a total of 38 significant genes. Detailed genetic information can be found in Supplementary Table S1.

### 3.4 Gene Ontology and Kyoto Encyclopedia of Genes and Genomes pathway enrichment analysis

Import the filtered 38 genes into the Metascape database. Perform GO enrichment analysis, which includes biological processes, molecular functions, and cellular components, as well as KEGG pathway enrichment analysis separately. Set the species to “sapiens” and the *p*-value threshold to less than 0.01. After that, conduct custom analysis and visualize the enrichment results using online bioinformatics graphing tools.

### 3.5 Machine learning for diagnostic signature genes

In the 10-fold cross-validation process, a total of 12 machine learning algorithms were combined: LASSO, Ridge, Stepglm, XGBoost, Random Forest (RF), Elastic Net (Enet), Partial Least Squares Regression for Generalized Linear Models (plsRglm), Generalized Boosted Regression Modeling (GBM), Naive Bayes (NB), Linear Discriminant Analysis (LDA), Generalized Linear Model Boosting (glmBoost), and Support Vector Machine (SVM), Through arrangement, 113 combinations are formed, and model development is carried out on the basis of 10-fold cross-validation, to determine the most powerful diagnostic model based on 38 intersecting genes. The model with a higher AUC value and good gene selection effect was ultimately chosen. Subsequently, the R package “pROC” was used for visualizing the receiver operating characteristic (ROC) curves of the training set and validation set.

### 3.6 Construction and independent validation analysis of model

The external dataset GSE165512 was used to validate the effectiveness of models GSE87466, GSE87473, and GSE92415 in distinguishing between ulcerative colitis (UC) and normal controls. AUC values for the selected genes were obtained using the R package “pROC” (version 1.18.5). A bar-line chart model was then created using the R package “rms” (version 6.3.0), where each predictor is assigned a score. The total score is the cumulative sum of the individual scores assigned to each predictor in the bar chart. Additionally, we used calibration curves and decision curve analysis (DCA) to evaluate the predictive capability of the bar chart.

### 3.7 Single-cell sequencing analysis

Download the GSE214695 data file from the NCBI GEO public database and use it for single-cell correlation analysis. Use the Seurat package to process the data, conduct PCA analysis, and perform clustering, dimensionality reduction, and cell visualization with the t-SNE algorithm and UMAP (uniform manifold approximation and projection). Next, annotate the cell clusters with the Celldex package. Finally, we extract the marker genes for each cell subtype from the single-cell expression profile using the logfc threshold and the FindAllMarkers function. Select the immune-related genes screened out by machine learning as the individual marker genes for each cell subtype.

### 3.8 Cell culture and establishment of ulcerative colitis models

Colon mucosal epithelial cells (CP-H040) were purchased from Pricella and cultured in RPMI-1640 (Gibco; 21870084) complete medium supplemented with 10% fetal bovine serum (FBS), 1% glutamine (Gibco; 25030081), and 1% penicillin-streptomycin (Gibco; 15070063) at 37°C in a 5% CO_2_ incubator. LPS solution was dissolved in the above complete medium and the LPS concentration was adjusted to 5 μg/mL. When the cell density reached 80%, the cells were washed twice with PBS, and the PBS was discarded. The cells were then exposed to RPMI-1,640 complete medium containing 5 μg/mL LPS and cultured for another 24 h. After the culture was completed, the cells were washed with PBS and RNA was extracted using RNA isolater Total RNA Extraction Reagent (Vazyme; R401-01) solution.

### 3.9 q-PCR analysis of UC-related markers

According to the manufacturer’s product instructions, RNA was extracted from cells using RNA isolater total RNA extraction reagent. The purity and concentration of RNA were evaluated using a microplate reader. The concentrations were adjusted to be consistent between the two groups. Reverse transcription was performed using the ABScript II cDNA First-Strand Synthesis Kit (ABclonal; RK20400). Real-time quantitative PCR reactions were conducted in a fluorescence quantitative PCR instrument (bio-rad; CFX Connect) in the presence of 2X Universal SYBR Green Fast qPCR Mix (ABclonal; RK21203). The reaction conditions were as follows: 95°C for 30 s for pre-denaturation; then 95°C for 15 s (denaturation), 60°C for 30 s (annealing/extension), for 40 cycles. The relative gene expression levels were calculated using the 2^−ΔΔCT^ method, with GAPDH as the internal control. All primers used in the study are listed in Supplementary Table S2.

### 3.10 Gene set enrichment analysis

Gene set enrichment analysis (GSEA) is used to elucidate the biological significance of characteristic genes by employing the fast GSEA R package. To achieve normalized enrichment scores for each analysis, gene set permutations are performed 1,000 times. False discovery rate (FDR) value <0.05 is considered significant enrichment.

### 3.11 Evaluation of the abundance and expression differences of immune cell subsets

Analyze the abundance and correlation of immune infiltrating cells using the CIBERSORT deconvolution algorithm. The CIBERSORT method mainly relies on the LM22 immune cell subtype expression matrix, using the CiberSortR script to perform qualitative and quantitative analysis on its matrix. Using the results from CIBERSORT, analyze the correlation between specific genes and immune cell types. Apply a screening criterion of *p* < 0.05 and utilize the corrplot package to create the corresponding plot (22).

### 3.12 Identification of candidate drugs

To identify candidate drugs for the pathological mechanisms potentially targeting ulcerative colitis (UC), we utilized the Drug Signatures Database (DSigDB) within the Enrichr network platform^2^ to screen for drugs. After identifying potential drugs, we filtered out those with high toxicity or significant side effects as reported in the literature, leaving us with 10 candidates for further analysis.

### 3.13 Molecular docking validation

Molecular docking is a computational method used to predict the docking pose and binding affinity between a protein and a ligand. To perform molecular docking, download the 3D structures of active compounds from the ChemSpider database.^3^ Then, use Chem3D to energy minimize the compounds and save them in .pdb format. The protein crystal structures of core genes CCL18 (Alphafold ID: AF-P55774-F1-v4), DUOX2 (Alphafold ID: AF-E7FEH3-F1-model_v4), GREM1 (Alphafold ID: AF-O60565-F1-v4), LCN2 (Alphafold ID: AF-P80188-F1-v4), and TNC (Alphafold ID: AF-P24821-F1-v4) were obtained from the Alphafold database.^4^ After adding hydrogen atoms, protons, and calculating charges using Autodock, both the receptor protein and the selected compounds are saved in PDBQT files. The position of the molecular docking binding site is optimized by modifying the center coordinates and size of the docking protein. Finally, Autodock Vina is used to dock the core receptor protein with the small molecule complex ligand. Each molecular docking generates 100 conformations, and the best docking conformation is selected based on binding energy and site as the final docking result. Lower binding energy indicates a stronger affinity between the key target and the core active ingredient. Subsequently, the interaction between the compound and protein is visualized using PYMOL software. In addition, the docking results are compared with reference drugs/inhibitors to assess reliability.

### 3.14 Molecular dynamics simulation

Molecular dynamics (MD) simulation of ligand-receptor docked complex was carried out using GROMACS (version 2022.2). Protein topology file was generated using the AMBER99SB-ILDN force field, whereas ligand topology file was generated by ACPYPE script using the AMBER fore field. We performed the MD simulation in a triclinic box filled with TIP3 water molecules, applying periodic boundary conditions. System was neutralized with NaCl counter ions. Before the MD simulation, we minimized the complex for 1000 steps and equilibrated it by running NVT and NPT for 100 ps. Then MD simulation was performed for 100 ns for each system under periodic boundary conditions at 310 K temperature and 1.0 bar pressure.

## 4 Discussion

UC is a chronic disease that affects the colonic mucosa. The global prevalence and incidence of ulcerative colitis have been rising over time. Notably, up to 15% of patients initially present with acute disease (23–25). Patients with ulcerative colitis face an increased risk of developing rectal cancer, which also imposes a significant economic burden on society (26). Currently, endoscopic examination and biopsy are the only methods for diagnosing ulcerative colitis (25). There are multiple treatments for this disease, with clinical practice primarily relying on drug therapy, such as 5-aminosalicylic acid (5-ASA), corticosteroids, immunosuppressants, and biologics. However, these medications have limitations, including side effects, individual differences, compliance issues, and economic burdens. No ideal treatments exist for ulcerative colitis in clinical practice, highlighting the urgent need for research into better therapeutic options (27).

Recent studies have found that the occurrence of ulcerative colitis (UC) is closely linked to immune function. I UC is a chronic and relapsing disease that occurs when genetically susceptible individuals have an abnormal immune response to intestinal luminal antigens (28). Several components of the mucosal immune system contribute to the pathogenesis of UC. These components include luminal antigens, intestinal epithelial cells (IEC), cells of the innate and adaptive immune systems, etc. (28). Additionally, research using mouse models has shown that the absence of certain immune cytokines significantly affects the likelihood of developing UC under various microbial conditions (29–31). Moreover, after treatment with broad-spectrum antibiotics, the inflammatory conditions of the experimental animals were alleviated (32). The intestine functions as a part of the innate immune system and has three lines of defense: the mucous layer, the epithelium, and the lamina propria, all of which contain a diverse range of immune cells that contribute to immune responses. When these barriers are damaged, immune tolerance is affected (33). Therefore, exploring the pathogenesis of UC from an immunological perspective and seeking potential therapeutic drugs is very meaningful.

Bioinformatics integrates biology, mathematics, and information science, employing computational methods to process, analyze, and interpret biological data like genomic sequences, protein structures, and metabolic pathways. Its strength lies in efficiently extracting insights using algorithms and software, aiding disease research. By analyzing gene expression, it identifies disease biomarkers, advancing personalized medicine and treatment strategies (34).

Through machine learning methods and experiments, we proved that the key genes: CCL18, DUOX2, GREM1, LCN2, and TNC are of great significance for the diagnosis and treatment of UC. CCL18 is a chemokine protein that primarily regulates the immune system. It regulates the migration and activation of white blood cells within the body by binding to its receptors. Recent studies have found that the CCL18 gene is located downstream of UC lncRNA, involved in the inflammatory immune response process of UC, and this process can be regulated by methylation (35–37). Comprehensive analyses of gene expression profiles in UC patients have confirmed that DUOX2 holds significant diagnostic and predictive value for mild to moderate UC, DUOX2 is a multifunctional enzyme that primarily participates in the synthesis of thyroid hormones and local immune defense, playing a significant role in immune responses and local cell signaling (38), it is a key enzyme in the production of hydrogen peroxide (H_2_O_2_) in the human colon, and its expression increases in active colitis as a response to inflammation (39). GREM1 is a gene that encodes a bone morphogenetic protein antagonist, belonging to the negative regulators of the bone morphogenetic protein (BMP) signaling pathway. Current experimental models suggest that the physiological attenuation of BMP signaling after ulceration can be promoted by upregulating the secretion of the antagonist GREM1 from different populations of fibroblasts, which in turn facilitates the regeneration of intestinal epithelial cells (40). Furthermore, this gene has been identified as a reliable potential biomarker for the diagnosis and treatment of UC. This biomarker mediates an immune response that is crucial for the onset and progression of UC by interacting with infiltrating immune cells (41). LCN2 is a key protein involved in iron metabolism, immune responses, kidney function, inflammation, and cancer development. Studies have found that LCN2 is a characteristic gene in the colonic mucosa of UC patients, and LCN2 plays a pro-inflammatory role in UC by promoting colonic epithelial cell pyroptosis, which may become a new target for inhibiting pyroptosis in UC. In addition, some studies suggest that LCN2 is a key factor in the regulation of ferroptosis in UC, providing more evidence for the significant role of ferroptosis in UC (42, 43). TNC (Tenascin C) is a multifunctional extracellular matrix protein involved in intercellular signaling and the maintenance of tissue structure and function. Its aberrant expression is associated with the development of various diseases. Studies have found that compared to normal controls, the expression level of TNC in the inflamed mucosa of UC patients is significantly elevated, and TNC is mainly expressed in the stromal areas of the intestinal mucosa. Studies also indicate that in UC patients treated with infliximab, the mucosal expression of TNC mRNA is higher and is associated with a poor response to treatment. This suggests that TNC may play a role in the pathogenesis of UC, especially in affecting patients’ responses to anti-TNF-α therapy. Therefore, the elevation of TNC may not only be a marker of inflammation in UC but could also serve as a biomarker for disease activity and response to treatment (44).

This study found that the expression characteristics of chemokine CCL18 and cytokinesTNF-α and IL-1β in the colonic tissues of UC patients suggest that they may become new therapeutic targets. CCL18 belongs to the CC subfamily of chemokines and exhibits complex dual roles in immune regulation. Its functional mechanisms show significant differences under different pathological and physiological conditions. Research indicates that CCL18 mediates immune cell migration via classical chemokine receptors, promoting NK cell movement and cytotoxicity in non-allergic individuals, while this function is impaired in allergic patients, indicating a specific imbalance in immune regulation (45, 46).

GO enrichment analysis revealed that the immune mechanism of UC is related to cytokine pathways, and pro-inflammatory cytokines TNF-α play an important role in the pathogenesis of UC. During UC, levels of TNF-α and other pro-inflammatory cytokines rise abnormally, leading to inflammatory responses. These inflammatory responses lead to damage of the intestinal mucosa, resulting in ulcers, bleeding and other symptoms, and aggravating the condition of UC. Studies have shown that metformin can alleviate DSS-induced colitis, and one of its mechanisms is that it inhibits the production of pro-inflammatory cytokines including TNF-α, thereby alleviating intestinal inflammation and further indicating that TNF-α has a close association with ulcerative colitis and inhibiting the production or activity of TNF-α may become a potential strategy for treating UC (47). Interleukin-1β (IL-1β) is also an important pro-inflammatory cytokine, which plays an important pro-inflammatory role in UC. IL-1β mainly promotes inflammatory responses by activating the NLRP3 inflammasome. In UC patients, activation of the NLRP3 inflammasome leads to the massive production of IL-1β, which recruits immune cells to the intestinal mucosa and triggers inflammatory responses. In addition, IL-1β can induce the differentiation of Th17 cells, which further secrete pro-inflammatory factors such as IL-17, exacerbating intestinal inflammation (48), indicating that this cytokine plays a key role in the pathogenesis of UC.

Single-cell analysis showed that key immune-related genes were highly expressed in macrophages, fibroblasts, and epithelial cells. Macrophages, as the core effector cells of innate immunity, their abnormal activation is a key mechanism for the uncontrolled inflammation in UC, and excessive activation can trigger persistent inflammatory responses, leading to symptoms such as diarrhea and abdominal pain (49). In UC, fibroblasts exhibit dynamic regulation. The reduction of pericanal fibroblasts is linked to goblet cell depletion and changes in the mucosal villi, which may worsen barrier disruption and increase the risk of carcinogenesis (50). In contrast, ulcer-related myofibroblasts help regulate the inflammatory microenvironment by secreting IL-33 and play a role in mucosal repair or fibrosis (51). Abnormal regulation of colonic epithelial cell apoptosis is an important feature of UC progression, and patients show significantly upregulated anti-apoptotic genes, resulting in epithelial barrier dysfunction and persistent inflammation (52). Therefore, the imbalance of macrophage activation, the heterogeneity of fibroblast phenotypes, and the dysregulation of epithelial cell apoptosis jointly drive the pathological process of UC.

The immune infiltration analysis indicates that UC is closely related to mast cells and neutrophils. Some studies have emphasized the significant activation role of mast cells during the active phase of UC. This study compared the number of mast cells in the active and remission phases of the same UC patient using immunohistochemical methods and found that the accumulation of mast cells in the colonic mucosa during the active phase was significantly higher than that in the remission phase (*p* < 0.01). Further analysis revealed that the number of mast cells in UC patients was even higher than that in colon cancer tissues, suggesting its specific role in inflammation (53). Some studies have revealed that the level of anti-neutrophil cytoplasmic antibody (ANCA) in the serum of UC patients is significantly elevated. The specificity of this is due to the binding of immunoglobulin G (IgG) of neutrophils and the nuclear perinuclear staining pattern. The high titer of ANCA in UC is associated with active inflammation and is distinct from other colitis and diarrhea diseases, indicating that it is not merely a concomitant phenomenon of inflammation, but an autoimmune reaction specific to the antigens of neutrophils. The combination of perinuclear ANCA and ELISA positive results has a high diagnostic specificity for UC (94%), suggesting that neutrophils may play a key role in the pathogenesis of UC (54).

To further accelerate the process of clinical treatment for UC, and to screen drugs for treating UC, we analyzed UC-related pathogenic genes through the DSigDB database to discover potential compounds targeting UC. Select 10 small molecule compounds (beclomethasone, ibuprofen, glycoprotein, simvastatin, budesonide, methotrexate, prednisolone, troglitazone, sulfasalazine, thalidomide) as candidate compounds. Beclomethasone is a corticosteroid commonly used to treat inflammation. It exerts its anti-inflammatory effect by binding to glucocorticoid receptors within cells. It reduces inflammation by inhibiting related molecules, such as cytokines and chemokines. Additionally, it helps suppress excessive immune reactions, thereby reducing damage caused by these responses. Beclomethasone has high receptor affinity and fewer side effects, making it commonly used for asthma and topical applications. Recently, it has also been found effective in treating patients with mild to moderate ulcerative colitis. Encapsulating it into fibrous micelles can greatly improve the utilization rate, which is reduced due to its hydrophobicity (55–57). Ibuprofen, a nonsteroidal anti-inflammatory drug (NSAID), inhibits cyclooxygenase (COX) to suppress prostaglandin synthesis, thereby alleviating pain, fever, and inflammation. Experimental studies demonstrate that ibuprofen specifically blocks inflammation-induced Rac1b upregulation in murine colon models (58), underscoring its therapeutic relevance in UC. Glycoproteins are a class of important biomolecules composed of proteins and sugar moieties (polysaccharides) linked by covalent bonds. Glycoproteins play critical roles in cell recognition, signal transduction, immune responses, and intercellular interactions. Glycoprotein is an important component of the intestinal epithelium and may play a role in the pathogenesis of intestinal inflammation, such as inflammatory bowel disease. Studies have found that ISO (isotope) can regulate intestinal microbiota and their metabolites, increase the expression of glycoprotein in the intestinal epithelium, thereby maintaining colonic homeostasis, improving the integrity of the colonic epithelium, and alleviating colitis (59). Simvastatin is a commonly used cholesterol-lowering drug belonging to the statin class of medications. Studies have shown that immune-mediated mechanisms play a dominant role in the action of statins. For instance, statins can weaken the activation and proliferation of T cells, thereby suppressing the secretion of pro-inflammatory cytokines and enhancing the release of anti-inflammatory cytokines (60). Recent mouse studies have shown that simvastatin can effectively treat inflammatory bowel disease by inhibiting NF-κB-induced IL-8 gene expression and blocking the transcriptional activity, phosphorylation, and DNA binding of NF-κB. Through analyses of body weight, colon length, disease activity index (DAI), and histology, simvastatin exhibited significant dose-dependent anti-inflammatory effects. These results suggest that simvastatin may attenuate the pathological process of inflammatory bowel disease (IBD) by modulating the NF-κB signaling pathway and suppressing the expression of pro-inflammatory genes, indicating its potential as a therapeutic agent for IBD (61). Budesonide is a steroid medication mainly used to treat inflammatory diseases. As a corticosteroid, budesonide has local anti-inflammatory effects. Studies show that its different formulations can serve various roles in treating UC (62), such as oral budesonide-MMX for the treatment of active, mild to moderate UC patients, and budesonide rectal foam for active, mild to moderate distal UC patients (from the anal margin to 40 centimeters) (63). Prednisolone is also a steroid medication that reduces inflammation and immune responses by inhibiting certain parts of the immune system, and Research indicates that budesonide has fewer side effects than prednisolone, making it a more favorable option in managing inflammatory bowel disease (64). Methotrexate (MTX) is an antimetabolite drug widely used to treat various cancers and certain immune system disorders. It works by inhibiting folate metabolism, disrupting DNA synthesis, and preventing cell division, which helps suppress tumor cell growth and the immune system’s overreaction. Currently, there is limited data on the use of Methotrexate in the treatment of UC. A study involving 40 patients treated with MTX for induction therapy found that it is safe and effective for maintaining clinical remission in those with UC in the short term (65). Troglitazone is an early drug used to treat type 2 diabetes, but a large number of studies in the literature have confirmed the beneficial effects of troglitazone in the intestine, with significant anti-inflammatory properties that can significantly reduce the levels of pro-inflammatory cytokines, thus potentially successfully used in the treatment and prevention of non-specific intestinal diseases (66, 67). Sulfasalazine is composed of sulfapyridine and 5-aminosalicylic acid (5-ASA), and is a widely used anti-inflammatory drug for the treatment of autoimmune diseases such as UC, Crohn’s disease, and rheumatoid arthritis (68). Sulfapyridine mainly reduces systemic inflammation by inhibiting the synthesis of inflammatory mediators, decreasing white blood cell infiltration at the inflammation site, and regulating cytokine secretion. In contrast, 5-ASA offers local protection to the intestinal mucosa by clearing oxidative free radicals, inhibiting neutrophil infiltration, and preserving the mucosal barrier’s integrity (69, 70). Thalidomide is a potent immunomodulatory drug used to treat various autoimmune diseases (71). Studies indicate that thalidomide can increase the mRNA expression of the anti-inflammatory factor IL-10 while decreasing the mRNA levels of pro-inflammatory factors IL-6, IL-1β, and TNF-α. Additionally, thalidomide exerts anti-inflammatory effects by inhibiting the PI3K/Akt pathway, which may explain its use in treating UC (72). The above-mentioned drugs, by targeting the immune mechanism of UC, provide a potential new strategy for the immunomodulatory treatment of ulcerative colitis and offer a reference for the development of new drugs for treating UC.

However, this article has certain limitations. In the future, adding more ulcerative colitis (UC) samples can optimize the machine learning model and improve its predictive accuracy. Although this article has identified potential drugs for treating ulcerative colitis, clinical trials have not yet been conducted, so their effectiveness in real-world settings cannot be determined. Thus, conducting relevant clinical trials is essential to verify the efficacy of these drugs.

## 5 Conclusion

This study employed various techniques and the experimental verification to analyze immune-related markers and immune-related mechanisms in UC. The identified immune biomarkers (CCL18, DUOX2, GREM1, LCN2, and TNC) demonstrated strong diagnostic efficacy and are key immune genes for ulcerative colitis (UC). Enrichment analysis revealed that DFU is related to inflammatory responses, leukocyte chemotaxis, etc. Additionally, we discovered potential therapeutic drugs, thalidomide and troglitazone. These findings enhanced the understanding of the pathogenesis of DFU and laid a solid foundation for future research and clinical applications, aiming to improve the prevention, diagnosis, and treatment of this severe diabetic complication.

## Data Availability

Publicly available datasets were analyzed in this study. This data can be found here: the original dataset utilized in this study was obtained from the Gene Expression Omnibus (GEO) database (https://www.ncbi.nlm.nih.gov/geo/), with the specific accession number and detailed data processing methodology comprehensively described in the Methods section.

## Footnotes

^1^ https://www.immport.org/home

^2^ https://amp.pharm.mssm.edu/enrichr/

^3^ https://www.chemspider.com/

^4^ https://alphafold.com/

## Glossary

RF: Random forest
LASSO: Least absolute shrinkage and selection operator
UC: Ulcerative colitis
WGCNA: Weighted gene co-expression network analysis
CIBERSORT: Cell-type identification by estimating relative subsets of RNA transcript
ROC: Receiver operating characteristic
GEO: Gene Expression Omnibus
DEGs: Differentially expressed genes
RMSD: Root mean square deviation
RMSF: Root mean square fluctuation
Rg: Radius of gyration
PCA: Principal component analysis
GO: Gene Ontology
KEGG: Kyoto Encyclopedia of Genes and Genomes
DCA: Decision curve analysis
GSEA: Gene set enrichment analysis
CCL18: C-C motif chemokine ligand 18
DUOX2: Dual oxidase 2
GREM1: Gremlin 1
LCN2: Lipocalin 2
TNC: Tenascin C
Enet: Elastic Net
GBM: Generalized boosted regression modeling
LDA: Linear discriminant analysis
SVM: Support vector machine
FDR: False discovery rate
DSigDB: Drug Signatures Database
MD: Molecular dynamics
5-ASA: 5-Aminosalicylic acid
NSAID: Nonsteroidal anti-inflammatory drug
IBD: Inflammatory bowel disease
DAI: Disease activity index
q-PCR: Quantitative polymerase chain reaction
